# Exploring the global geography of cybercrime and its driving forces

**DOI:** 10.1057/s41599-023-01560-x

**Published:** 2023-02-23

**Authors:** Shuai Chen, Mengmeng Hao, Fangyu Ding, Dong Jiang, Jiping Dong, Shize Zhang, Qiquan Guo, Chundong Gao

**Affiliations:** 1 Institute of Geographic Sciences and Nature Resources Research, Chinese Academy of Sciences, Beijing, China; 2 College of Resources and Environment, University of Chinese Academy of Sciences, Beijing, China; 3 Big Data Center of State Grid Corporation of China, Beijing, China; 4 The Administrative Bureau of Chinese Academy of Sciences, Beijing, China

**Keywords:** Science, technology and society, Geography, Criminology

## Abstract

Cybercrime is wreaking havoc on the global economy, national security, social stability, and individual interests. The current efforts to mitigate cybercrime threats are primarily focused on technical measures. This study considers cybercrime as a social phenomenon and constructs a theoretical framework that integrates the social, economic, political, technological, and cybersecurity factors that influence cybercrime. The FireHOL IP blocklist, a novel cybersecurity data set, is used to map worldwide subnational cybercrimes. Generalised linear models (GLMs) are used to identify the primary factors influencing cybercrime, whereas structural equation modelling (SEM) is used to estimate the direct and indirect effects of various factors on cybercrime. The GLM results suggest that the inclusion of a broad set of socioeconomic factors can significantly improve the model’s explanatory power, and cybercrime is closely associated with socioeconomic development, while their effects on cybercrime differ by income level. Additionally, results from SEM further reveals the causal relationships between cybercrime and numerous contextual factors, demonstrating that technological factors serve as a mediator between socioeconomic conditions and cybercrime.

## Introduction

Cybercrime is a broad term used by government, businesses, and the general public to account for a variety of criminal activities and harmful behaviours involving the adoption of computers, the internet, or other forms of information communications technologies (ICTs) (Wall, 2007). As an emerging social phenomenon in the information age, cybercrime has aroused growing concern around the world due to its high destructiveness and widespread influence. In 2017, the WannaCry ransomware attack affected more than 230,000 computers across 150 countries, resulting in economic losses of more than 4 billion dollars and posing a serious danger to the global education, government, finance, and healthcare sectors (Ghafur et al., 2019; Castillo and Falzon, 2018; Mohurle and Patil, 2017). Although there is currently no precise and universally accepted definition of cybercrime (Phillips et al., 2022; Holt and Bossler, 2014), it is generally acknowledged that the term covers both traditional crimes that are facilitated or amplified by utilising ICTs as well as new types of crimes that emerged with the advent of ICTs (Ho and Luong, 2022). Based on the role of technology in the commission of the crime, the most widely utilised typology divides cybercrime into cyber-dependent crime (such as hacking, distributed denial of service, and malware) and cyber-enabled crime (online fraud, digital piracy, cyberbullying) (Brenner, 2013; Sarre et al., 2018; McGuire and Dowling, 2013). Along with the rapid development of ICTs and the increasing prevalence of the internet, these criminal activities are significantly disrupting the global economy, national security, social stability, and individual interests. Although it is difficult to estimate the precise financial cost of cybercrime (Anderson et al., 2013; Anderson et al., 2019), statistical evidence from governments and industries indicates that the economic losses caused by cybercrime are extremely enormous and are still rising rapidly (McAfee, 2021).

Cybercrime is complicated in nature and involves many disciplines, including criminology, computer science, psychology, sociology, economics, geography, political science, and law, among others (Holt, 2017; Dupont and Holt, 2022; Payne, 2020). Computer science and cybersecurity efforts are primarily focused on applying technical approaches such as Intrusion Detection Systems (IDSs), Intrusion Prevention Systems (IPSs), firewalls, and anti-virus software to mitigate cyberattack threats (Kumar and Carley, 2016; Walters, 2015). These methods may help to some extent lessen the adverse impacts of cybercrime on both organisations and individuals. However, these technical solutions are largely unaware of the human and contextual factors that contribute to the issues, providing only reactive solutions, and are unable to keep up with the rapidly evolving *modus operandi* and emerging technologies (Clough, 2015; Neal, 2014). It is suggested that cybercrime is a complex social phenomenon driven by the compound interactions of underlying socioeconomic factors. Human and social factors play a substantial role in the formation of cybercrime agglomerations (Waldrop, 2016; Watters et al., 2012; Leukfeldt and Holt, 2019). They are also important aspects of cybercrime prevention and control (Dupont and Holt, 2022). The human factors influencing cybercrime have been the subject of an expanding body of sociological and psychological study in recent years. These studies, which covered cyberbullying, online harassment, identity theft, online fraud, malware infection, phishing, and other types of cybercrime, generally applied traditional criminological and psychological theories, such as routine activities theory, lifestyle-routine activities theory, self-control theory, and general strain theory, to explain the victimisation and offending of various cybercrimes (Bergmann et al., 2018; Mikkola et al., 2020; Ngo and Paternoster, 2011; Pratt et al., 2010; Williams, 2016). Results from these studies suggested that by altering criminal motivations and opportunity structures, individual factors (i.e., age, gender, ethnicity, education, socioeconomic status, and self-control) and situational factors (online activities, time spent online, risk exposure, deviant behaviours) may have an impact on cybercrime offence and victimisation. These findings advanced our knowledge in understanding the impact of technology on criminal behaviours, factors affecting the risk of cyber victimisation, and the applicability of traditional criminological theories to cybercrime (Holt and Bossler, 2014).

Cybercrime is a highly geographical phenomenon on a macro-level scale, with some countries accounting for a disproportionate amount of cybercrimes (Kigerl, 2012; Kigerl, 2016). This spatial heterogeneity is closely related to specific socioeconomic contexts (Kshetri, 2010). Academic efforts have been made to identify the clusters of high cybercrime countries and to explain the potential socioeconomic factors that led to the formation of these clusters. For example, Mezzour, Carley, and Carley (2014) found that Eastern European countries hosted a greater number of attacking computers due to their superior computing infrastructure and high levels of corruption. Similarly, Kumar and Carley (2016) found that higher levels of corruption and large internet bandwidth would favour attack origination. They also noted that countries with the greater gross domestic product (GDP) per capita and better ICT infrastructure were targeted more frequently. Meanwhile, Srivastava et al. (2020) pointed out that countries with better technology and economic capital were more likely to become the origins of cybercrime, but countries with better cybersecurity preparedness may reduce the frequency of the cybercrime originating within them. Moreover, Holt, Burruss, and Bossler (2018) suggested that nations with better technological infrastructure, greater political freedom, and fewer organised crime were more likely to report malware infections, while Overvest and Straathof (2015) suggested that the number of internet users, bandwidth, and economic ties were significantly related to cyberattack origin. Kigerl (2012) found that a higher unemployment rate and more internet users were linked to an increase in spam activities. However, these studies have tended to utilise a restricted range of predictor variables and only included certain aspects of cybercrime. Besides, most of the studies have been conducted at the national level, which could potentially hide many disparities within countries.

In this work, we construct a conceptual model to better represent the context from which cybercrime emerges, which is applied as a framework to analyse the underlying socioeconomic driving forces. A novel cybersecurity data set, the FireHOL IP blocklist, is adopted as a proxy to reflect the levels of cybercriminal activities within different areas. A set of social, economic, political, technological, and cybersecurity indicators is used as explanatory variables. Generalised linear models (GLMs) are used to quantify the effect of each factor on cybercrime, while structural equation modelling (SEM) is used to estimate the complex interactions among various factors and their direct and indirect effects on cybercrime.

## Conceptual framework

**Fig. 1:**
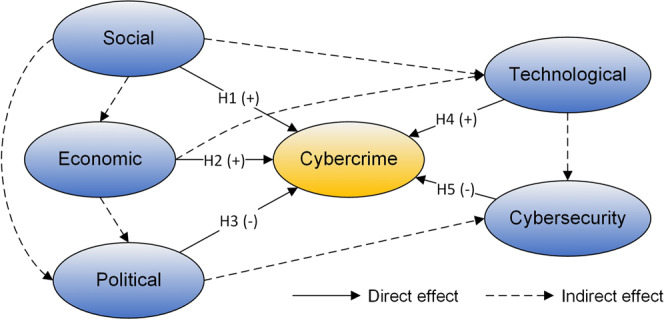
The conceptual framework for analysing driving forces of cybercrime. The solid line indicates a direct effect, and a dashed line indicates indirect effect. H1–H5 refer to the five hypotheses, “+” indicates a positive effect, and “−” indicates a negative effect.

We propose a conceptual framework for examining the driving forces of cybercrime by reviewing existing empirical literature and integrating different criminological theories. The conceptual framework includes five interrelated components: the social, economic, political, technological, and cybersecurity factors. The potential pathways by which each component may directly or indirectly influence cybercrime are illustrated in Fig. 1.

The social and economic factors depict the level of regional development, serving as the fundamental context in which cybercrime emerges. Given the intrinsic technological nature of cybercrime, global urbanisation, and the information technology revolution have promoted global connectivity and created unprecedented conditions and opportunities for cybercrime (UNODC, 2013). From the perspective of general strain theory, poverty, unemployment, income inequality, and other social disorders that are accompanied by social transformations could lead to cultures of materialism and stimulate motivations of cybercrime for illegal gains (Meke, 2012; Onuora et al., 2017). On the other hand, economically developed regions generally have superior ICT infrastructure, which can provide convenient and low-cost conditions for cybercriminals to commit crimes. High educational attainment is also likely to be associated with cybercrime, given that cybercrime usually requires some level of computer skills and IT knowledge (Holt and Schell, 2011; Asal et al., 2016). In general, better socioeconomic conditions are associated with more cybercriminal activities, which leads us to develop the first two hypotheses:

*H1: Social factor is positively associated with cybercrime*.

*H2: Economic factor is positively associated with cybercrime*.

The influence of political factors on cybercrime is mainly reflected in the regulation and intervention measures of governments in preventing and controlling cybercrime, such as legal system construction, government efficiency, control of corruption, and political stability. The offender’s decision to engage in illegal activity is a function of the expected probability of being arrested and convicted and the expected penalty if convicted (Ehrlich, 1996). As with traditional crimes, the lack of efficient social control and punishment mechanism will breed criminal behaviours. The deterrent effect of the legislation makes cybercriminals have to consider the consequences they need to bear. While the virtual and transnational nature of cyberspace makes it easier for perpetrators to avoid punishment, cybercrime can be deterred to some extent by increasing the severity of punishment and international law enforcement cooperation (Hall et al., 2020). On the other side, cybercriminals could seek protection through corrupt connections with the local institutional environment, which would weaken law enforcement operations and encourage cybercriminal activities (Hall et al., 2020; Lusthaus and Varese, 2021; Sutanrikulu et al., 2020). For instance, corruption in law enforcement authorities makes it hard for cybercriminals to be punished, while corruption in network operators or internet service providers (ISPs) makes it easier for cybercriminals to apply for malicious domain names or register fake websites. Some studies have shown that areas with high levels of corruption usually have more cybercriminal activities (Mezzour et al., 2014; Watters et al., 2012). Cybercrimes are typically attributed to political corruption, ineffective governance, institutional weakness, and weak rule of law across West Africa and East Europe (Asal et al., 2016). Therefore, we propose that:

*H3: Political factor is negatively associated with cybercrime*.

The technological environment, which is composed of communication conditions and underlying physical ICT infrastructure, serves as an essential medium through which cybercrime is committed. According to the rational choice theory, crime is the result of an individual’s rational consideration of the expected costs and benefits attached to their criminal activity (Mandelcorn et al., 2013; Brewer et al., 2019). Better internet infrastructure, greater internet penetration, and faster connection could facilitate cybercrimes by reducing crime costs, expanding opportunities, and increasing potential benefits. For example, in a majority of spam and DDoS attacks, cybercriminals often carry out large-scale coordinated attacks by sending remote commands to a set of compromised computers (also known as botnets). High-performance computers and high-bandwidth connectivity such as university, corporate, and government servers allow for more efficient attacks and could expand the scope of cybercrime, making them preferred by cybercriminals (Hoque et al., 2015; Van Eeten et al., 2010; Eslahi et al., 2012). We thus hypothesise that:

*H4: Technological factor is positively related to cybercrime*.

Cybersecurity preparedness reflects the capabilities and commitment of a country to prevent and combat cybercrime. According to the International Telecommunication Union (ITU), cybersecurity preparedness involves the legal, technical, organisation, capacity, and cooperation aspects (Bruggemann et al., 2022). Legal measures such as laws and regulations define what constitutes cybercrime and specify necessary procedures in the investigation, prosecution, and sanction of cybercrime, providing a basis for other measures. Technical measures refer to the technical capabilities to cope with cybersecurity risks and build cybersecurity resilience through national institutions and frameworks such as the Computer Incident Response Teams (CIRTs) or Computer Emergency Response Teams (CERTs). Organisation measures refer to the comprehensive strategies, policies, organisations, and coordination mechanisms for cybersecurity development. Capacity development reflects the research and development, awareness campaigns, training and education, and certified professionals and public agencies for cybersecurity capacity building. Cooperation measures refer to the collaboration and information sharing at the national, regional, and international levels, which is essential in addressing cybersecurity issues given the transnational nature of cybercrime. According to the general deterrence theory and routine activity theory of criminology (Leukfeldt and Holt, 2019; Hutchings and Hayes, 2009; Lianos and McGrath, 2018), cybersecurity preparedness serves as a deterrent or a guardianship of cybercrime. It is crucial in defending a country from external cybercrime as well as reducing cybercrime originating from within. Therefore, we hypothesise that:

*H5: Cybersecurity preparedness is negatively associated with cybercrime*.

The five hypotheses proposed in the conceptual model (Fig. 1) outline the direct effects of various contextual drivers on cybercrime. The social, economic, political, technological, and cybersecurity factors may interact in other ways, which could also have an indirect impact on cybercrime. Then, using a combination of two statistical methods and a set of explanatory covariates, we test the hypothesised pathways.

## Methods

### Data

#### Cybercrime data

It is commonly acknowledged among cybercrime scholars that the lack of standardised legal definitions of cybercrime and valid, reliable official statistics makes it difficult to estimate the prevalence or incidence of cybercrime around the world (Holt and Bossler, 2015). Although in some countries, law enforcement agencies do collect data on cybercrime (e.g., police data and court judgement), there are inevitable under-reporting and under-recording issues with these official data (Holt and Bossler, 2015; Howell and Burruss, 2020). This has prompted some researchers to use alternative data sources to measure cybercrime, including social media, online forums, emails, and cybersecurity companies (Holt and Bossler, 2015). Among these data sources, technical data such as spam emails, honeypots, IDS/IPS or firewall logs, malicious domains/URLs, and IP addresses are often used as proxies for different aspects of cybercrime (Amin et al., 2021; Garg et al., 2013; Kigerl, 2012; Kigerl, 2016; Kigerl, 2021; Mezzour et al., 2014; Srivastava et al., 2020; Kshetri, 2010), accounting for a large proportion in the literature of macro-level cybercrime research. However, due to the anonymity and virtuality of cyberspace, cybercriminals are not restrained by national boundaries and could utilise compromised computers distributed around the world as a platform to commit cybercrime. Meanwhile, IP addresses can be faked or spoofed by using technologies such as proxy servers, anonymity networks, and virtual private networks (VPNs) to hide the true identity and location of cybercriminals (Holt and Bossler, 2015; Leukfeldt and Holt, 2019). As a result, the attribution of cybercriminal becomes extremely challenging and requires a high level of expertise and coordination from law enforcement agencies and cybersecurity teams (Lusthaus et al., 2020). Therefore, instead of capturing where cybercriminals reside in physical space, most studies using these technical data are measuring the possible locations where the cyberattacks or cybercrimes originate, even if part of them could be locations where cybercriminals choose to host their botnets or spam servers. Though there is partial support that certain types of cyberattacks originate from physically proximate IP addresses (Maimon et al., 2015), more elaborate and comprehensive research is lacking.

In this study, we used a novel cybersecurity data set, the IP addresses from FireHOL blocklist (FireHOL, 2021), as a proxy to measure cybercrime. The FireHOL IP blocklist is a composition of multiple sources of illegitimate or malicious IP addresses, which can be used on computer systems (i.e., servers, routers, and firewalls) to block access from and to these IPs. These IPs are related to certain types of cybercrime activities, including abuse, attacks, botnets, malware, command and control, and spam. We adopt FireHOL level 1 blocklist, which consists of ~2900 subnets and over 600 million unique IPs, with a minimum of false positives. The anonymous IPs, which are used by other parties to hide their true identities, such as open proxies, VPN providers, etc., were excluded from the analysis. Next, we applied an open-source IP geolocation database, IP2Location™ Lite, to map these unique IP addresses in specific geographic locations in the form of country/region/city and longitude/altitude pair. The location accuracy of the IP geolocation is high at the national and regional levels, with ~98% accuracy at the country level and 60% at the city level. In order to reduce uncertainty, we focused on the analysis at the state/region level. At last, we calculated the counts of unique IPs located within each subnational area to measure the global distribution of cybercrimes.

Although FireHOL IP blocklist has the same restrictions as other technical data, it was used in this study for several reasons. The basic function of IP addresses in the modern internet makes it an indispensable element in different phases of cybercrime, it is also the key ingredient of cybercrime attribution and digital evidence collection. As a result, an IP-based firewall is one of the most effective and commonly used preventive measures for cybersecurity defence. FireHOL IP blocklist has the advantage of global coverage and includes different cybercrime types. It dynamically collects cybercrime IPs from multiple sources around the world. Although it is difficult to determine whether the IPs in the blocklist are the real sources of cybercrime or come from infected machines, it does reflect the geographical distribution of the malicious IPs that are related to certain cybercrime activities. Besides, it provides a more fine-grained estimate of the subnational cybercrime geography than country-level statistics.

#### Explanatory variables

We adopted a broad set of explanatory variables to characterise the social, economic, political, technological, and cybersecurity conditions based on the conceptual model presented above (Fig. 1). The social environment is represented by population, the population aged 15–64, education index, nighttime light index, and human development index (HDI); The economic condition is measured by income index, GDP growth, Gini index, unemployment (% of the total labour force) and poverty rate; The political environment is measure by 5 dimensions of the World Governance Indicators (WGI), including control of corruption, government effectiveness, rule of law, political stability and absence of violence/terrorism, voice and accountability. The technological environment is reflected by the internet infrastructure (the number of internet data centres and internet exchange centres), internet users (% of the population), international bandwidth (per internet user), secure internet server (per 1 million people), and fixed broadband subscriptions (per 100 people). Moreover, we applied the five dimensions of the Global Cybersecurity Index (GCI) to assess the level of commitment among various nations to cybersecurity, including legal measures, technical measures, organisational measures, capacity development measures, cooperation measures, and one overall cybersecurity index (the sum of the 5 measures above). Population, income index, education index, HDI, nighttime light, and infrastructure data are collected at the subnational administrative level, while other variables are derived at the country level. Log transformations (base 10) were used to improve normality for variables with skewed distributions, including population, nighttime light, infrastructure, fixed broadband, secure internet server, and bandwidth. All variables were normalised for further analysis.

### Models

#### Generalised linear models (GLMs)

In this study, GLMs were used to assess the potential influence of various explanatory variables on cybercrime and to identify the most important factors. A GLM is an extension of a regular regression model that includes nonnormal response distributions and modelling functions (Faraway, 2016). GLM analyses were conducted at two scales: the global scale and the income group scale. All GLMs were built in R version 4.1.2 using the “glm” function of the “stats” package (R, Core Team, 2013), and a gaussian distribution is used as the link function. The Akaike information criterion (AIC), the determination coefficient (*R*^2^), and the significance level of the predictors (*p*-value) are used to evaluate GLMs. The model with the lowest AIC and highest *R*^2^ value is chosen as the optimal model. Variance inflation factors (VIFs) were calculated using the “car” package (Fox et al., 2012) to test for collinearity between quantitative explanatory variables prior to the GLM analysis. Variables with a VIF value greater than 10 (VIF > 10) were regarded as collinearity generators and were therefore excluded from further analysis. The relative contribution and coefficients of each GLM were plotted using the “GGally” package.

#### Structural equation modelling (SEM)

SEM was used to examine the causal relationships within the networks of interacting factors, thereby distinguishing the direct from indirect drivers of cybercrime. SEM is a powerful, multivariate technique found increasingly in scientific investigations to test and evaluate multivariate causal relationships (Fan et al., 2016). SEM differs from other modelling approaches in that it tests both the direct and indirect effects on pre-assumed causal relationships. The following fit indices were considered to evaluate model adequacy: (a) root mean square error of approximation (RMSEA), which is a “badness of fit” index in which 0 indicates a perfect fit while higher values indicate a lack of fit; (b) standardised root mean square residual (SRMR), which is similar to RMSEA and should be less than 0.09 for good model fit; (c) comparative fit index (CFI), which represents the amount of variance that has been accounted for in a covariance matrix ranging from 0.0 to 1.0, with a higher CFI value indicating better model fit; (d) Tucker–Lewis index (TLI), which is a non-normed fit index (NNFI) that proposes a fit index independent of sample size. In this study, SEM analysis was conducted using AMOS (Arbuckle, 2011).

## Results

### Spatial distribution of cybercrime IPs

**Fig. 2:**
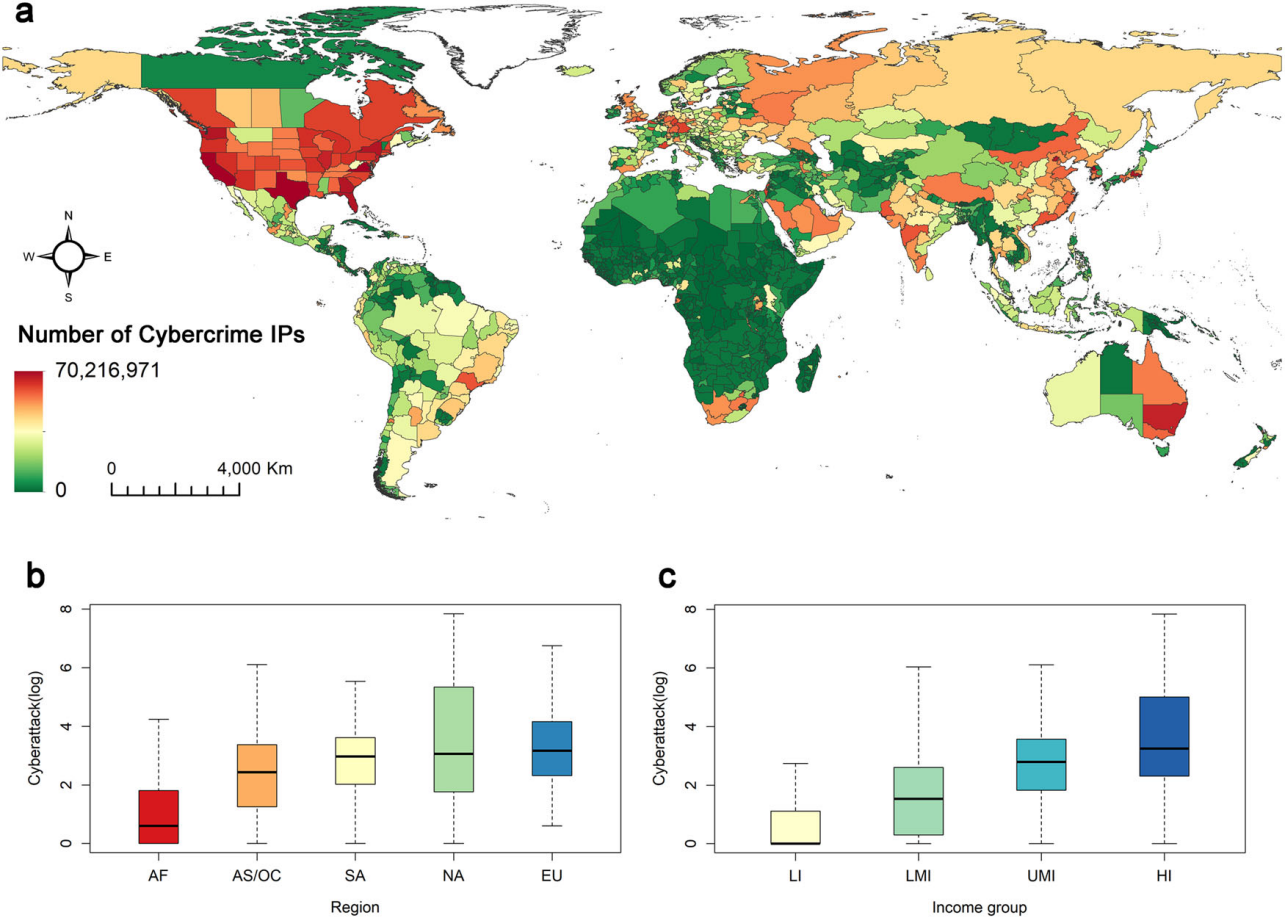
Global distribution of cybercrime IPs. **a** Number of cybercrime IPs at the subnational level. **b** Log-transformed cybercrime IP count by continent: Africa (AF), Asia/Oceania (AS/OC), South America (SA), North America (NA) and Europe (EU). **c** Log-transformed cybercrime IP count by income group: low-income (LI), lower-middle-income (LMI), upper-middle-income (UMI) and high-income (HI) groups. The centre line, boxes, and whiskers show the means, 1 standard error (SE), and 95% confidence interval (CI), respectively.

We mapped the subnational distribution of cybercrime IPs globally, which reveals significant spatial variability (see Fig. 2). On a global scale, most cybercrime IPs were located in North America, Central and Eastern Europe, East Asia, India, and eastern Australia. Meanwhile, areas with low numbers of cybercrime IPs were primarily found in large parts of Africa except for South Africa, western and northern parts of South America, Central America, some regions of the Middle East, southern parts of Central Asia, and some regions of Southeast Asia. On a continental scale, we found that the number of cybercrime IPs increased gradually from Africa to Europe. The two continents with the most cybercrime IPs were North America and Europe, with North America showing more variations. This trend seems to be closely associated with the regional socioeconomic development level. To further investigate this relationship, we grouped the subnational regions by income level according to the World Bank classification rules. We found a more evident pattern, with high-income regions hosting the majority of cybercrime IPs and lower-middle-income regions hosting the least.

### Major factors influencing cybercrime

**Fig. 3:**
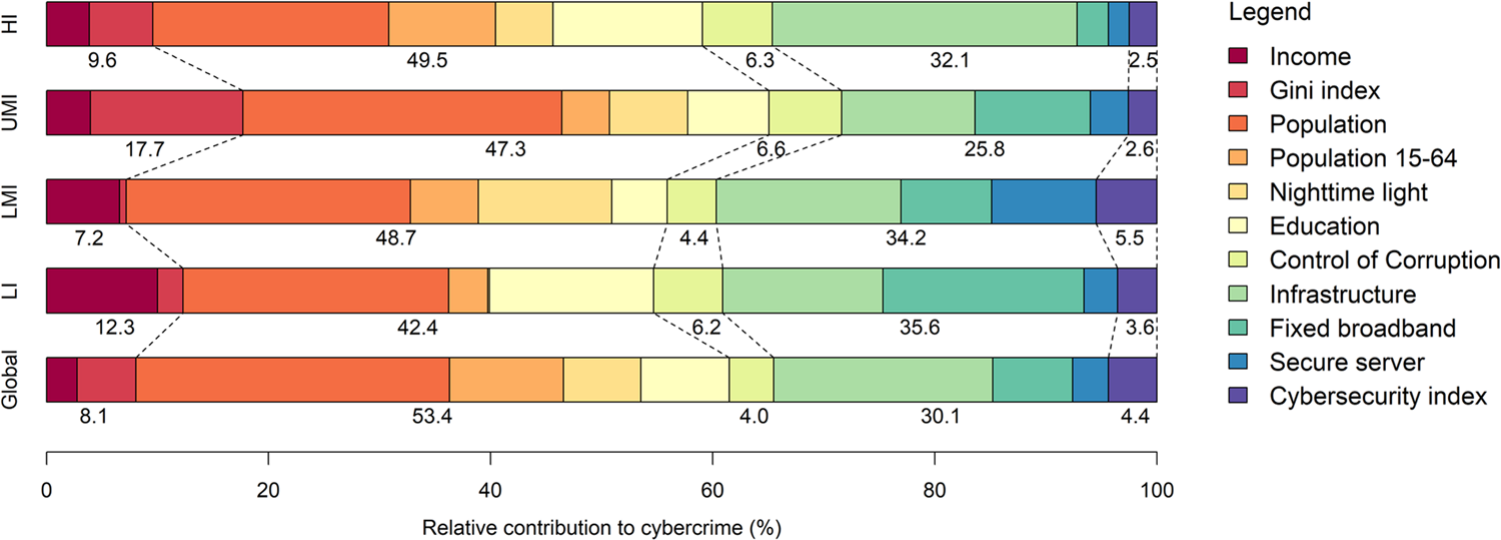
Relative contribution of predictor variables to cybercrime.

GLMs were built based on the 5 categories of 26 representative influential variables identified in the conceptual framework. After excluding 8 collinear variables (i.e., government effectiveness, rule of law, HDI, and 5 cybersecurity measures) and 7 nonsignificant variables (GDP growth, unemployment, poverty, political stability, voice and accountability, bandwidth, and internet users), the global scale GLM model includes 11 variables with an *R*^2^ value of 0.82. Figure 3 shows the relative contribution of each predictor variable to the model. Globally, the social and technological factors contribute most to the model, with relative contribution rates of 53.4% and 30.1%, respectively. Infrastructure alone explains up to 18.1% of the model variance in cybercrimes (*R*^2^ to 0.504). However, the inclusion of the population and education index improves the explanation of model variance by 18.3% and 28.5%, respectively (*R*^2^ to 0.596 and 0.766). This is also the case with GLMs of different income groups, indicating that despite the main effects of technological factors, the inclusion of a broad set of socioeconomic factors significantly improves the accuracy of models that attempt to quantify the driving forces of cybercrime.

When assessed by income group, we noted that although the social and technological factors were the most important factors in explaining cybercrime, the contribution of each variable varies by income group. For example, the contribution of the income index decreases gradually from low-income regions to wealthier regions, while the Gini index is more significant in upper-middle regions and high-income regions than in low-income regions and lower-middle-income regions. Fixed broadband subscriptions contributed the most in low-income regions and the least in high-income regions. Additionally, cybersecurity preparedness has a greater influence on low-income and lower-middle-income regions.

### Estimated effect of factors on cybercrime

**Fig. 4:**
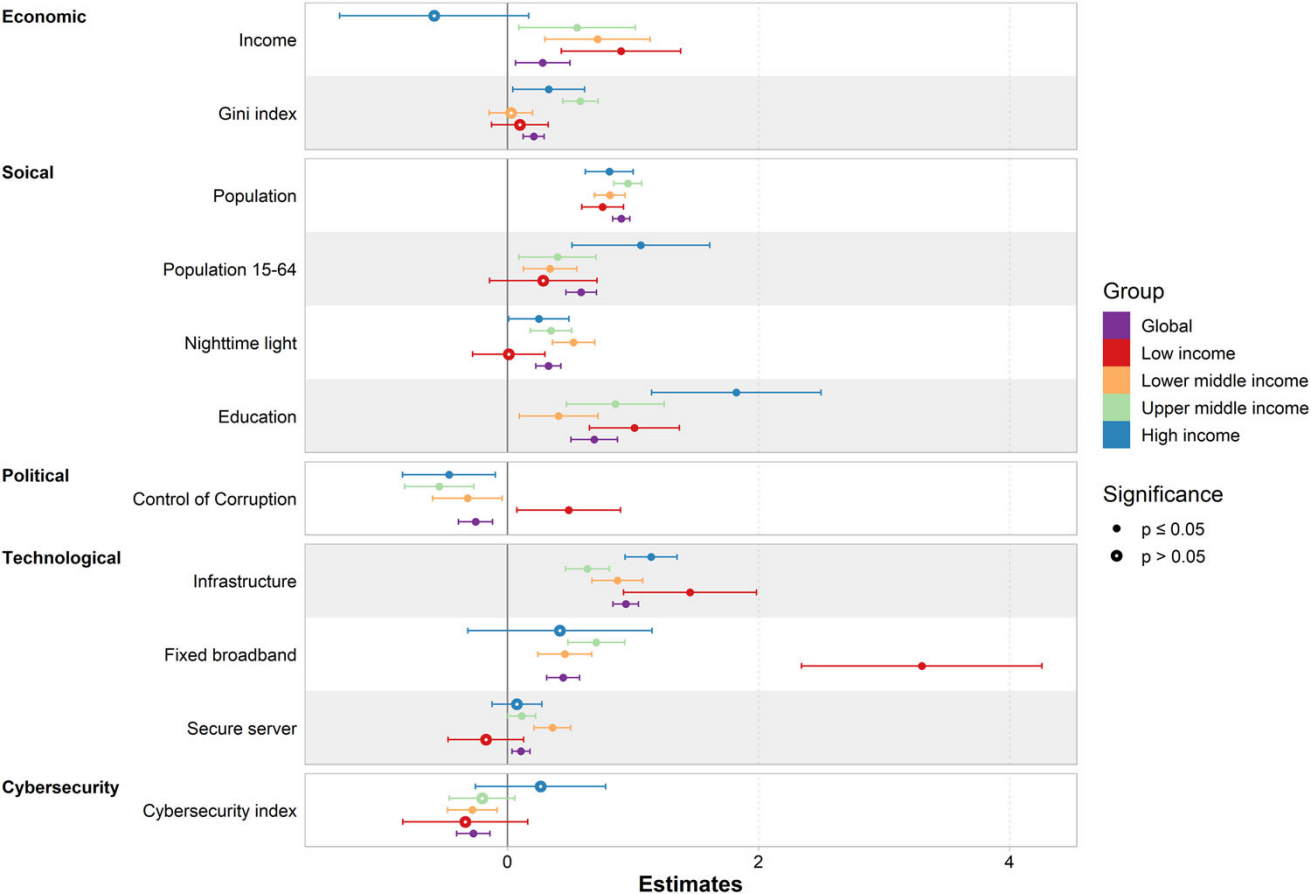
Effects of predictor variables on cybercrime. The coefficient values are represented as dots, significant variables are represented as filled dots, nonsignificant variables are represented as hollow dots, and bars represent 95% CIs.

The coefficient values in Fig. 4 represent effect sizes from the GLMs for the relationship between cybercrime and the five categories of contextual factors. At the global scale, cybercrime is positively correlated with social, economic, and technological factors, suggesting that most cybercrimes are launched in regions with a higher population, higher urbanisation, better educational and economic conditions, and, most importantly, improved internet infrastructure and communication conditions. By contrast, cybercrime is negatively related to political and cybersecurity factors, indicating that the control of corruption and the commitment to cybersecurity show certain inhibitory effects on cybercrime.

From the perspective of income groups, the ways contextual factors affect cybercrime remain basically consistent with the global results, but subtle differences are observed. In low-income countries, the influence of the income index on cybercrime is the strongest, and cybercrime is significantly associated with a higher income index, higher education index, better infrastructure, and higher fixed broadband subscriptions. This pattern may indicate that in low-income countries, wealthier areas tend to have more cybercrimes due to the existence of better communication conditions in these areas. However, in high-income countries, where the internet is universally available, the roles of income index and fixed broadband subscriptions gradually weaken. In contrast, the effects of the Gini index and education are stronger in wealthier countries, indicating that economic inequality and education in these countries can be important drivers of cybercrime. Moreover, the control of corruption is negatively related to cybercrime in lower-middle, upper-middle, and high-income regions.

### Pathways of factors for cybercrime

**Fig. 5:**
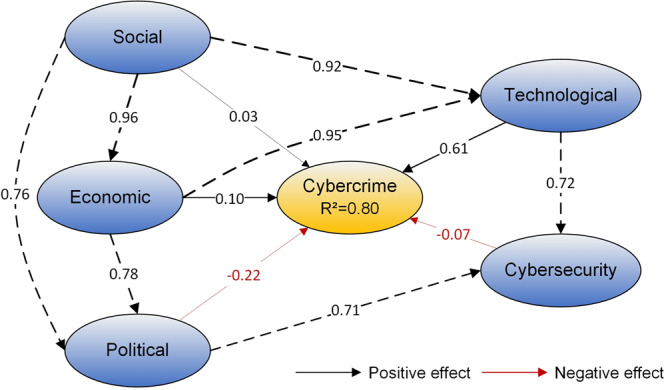
SEM path diagram for the effect of the driving forces on cybercrime. Black arrows indicate a positive effect, red arrows indicate a negative effect, and values on the straight arrows between variables represent the standardised path coefficients.

To understand the intricate interactions among different predictors, we perform SEM based on the conceptual model. The SEM model is composed of five latent variables, representing the social, economic, political, technological, and cybersecurity context, and each latent variable has five components reflected by the explanatory variables. Overall SEM fit is assessed, showing a good fit (CFI = 0.917, TLI = 0.899, SRMR = 0.058). SEM confirms many of the hypotheses in the conceptual model, and all relationships are statistically significant. Fig. 5 shows the results of SEM.

According to the SEM, all the hypotheses are tested and supported. Specifically, social, economic, and technological factors have direct positive effects on cybercrime (standardised path coefficients of direct effect are 0.03, 0.10, and 0.61, respectively), indicating that when social, economic, and technological factors go up by 1 standard deviation, cybercrime goes up by 0.03, 0.10, and 0.61 standard deviations, respectively. By contrast, the political and cybersecurity factors have direct negative effects on cybercrime (standardised path coefficients of direct effect are −0.22 and −0.07, respectively), indicating that 1 standard deviation rise in political and cybersecurity factors are associated with 0.22 and 0.07 standard deviations decrease of cybercrime, respectively. It is worth noting that although the direct effects of social and economic factors on cybercrimes are relatively small, their indirect effects on cybercrime through the mediation of technological and political factors are non-negligible.

In sum, SEM quantifies the direct and indirect effects of social, economic, political, technological, and cybersecurity factors on cybercrime, consistent with the hypotheses outlined in the conceptual model. More importantly, the results suggest that even though cybercrimes are primarily determined by technological factors, the direct and indirect effects of underlying social, economic, political, and cybersecurity also play significant roles. This suggests that the technological factor is a necessary but not sufficient condition for the occurrence of cybercrime.

## Discussion

In the current study, we mapped the global subnational distribution of cybercrimes based on a novel cybersecurity data set, the FireHOL IP blocklist. Given the widespread difficulty in obtaining cybercrime data, the data sources used in this study could provide an alternative measure of the subnational cybercrime level on a global scale. Compared to country-level studies (Amin et al., 2021; Garg et al., 2013; Goel and Nelson, 2009; Solano and Peinado, 2017; Sutanrikulu et al., 2020), the results present a more fine-grained view of the spatial distribution of cybercrime. The map reveals high spatial variability of cybercrime between and within countries, which appears to be closely related to local socioeconomic development status.

To recognise the driving forces behind cybercrime, we proposed a theoretical framework that encompasses the social, economic, political, technological, and cybersecurity factors influencing cybercrime, drawing on existing theoretical and empirical research. On this basis, we used GLMs to identify the major factors and their contributions to cybercrime and SEM to quantify the direct and indirect effects of these driving forces. The GLM results show that using technological factors alone as explanatory variables is insufficient to account for cybercrime, and the inclusion of a broad suite of social, economic, political, technological, and cybersecurity factors can remarkably improve model performance. Global scale modelling indicates that cybercrime is closely associated with socioeconomic and internet development, as developed regions have more available computers and better communication conditions that facilitate the implementation of cybercrime. Some studies have argued that wealthier areas might have fewer incentives for cybercrime, while poorer areas could benefit more from cybercrime activities (Ki et al., 2006; Kigerl, 2012; Kshetri, 2010). However, our study shows that the technological factors constituted by the internet infrastructure and communication conditions are necessary for the production of cybercrime, rendering wealthier areas more convenient for committing cybercrime.

Meanwhile, the GLMs of the 4 income groups demonstrate important differential impacts of the explanatory variables on cybercrime. For example, in low-income countries, where the overall internet penetration rate is low, cybercrime originates mainly in more developed areas with better internet infrastructure, higher internet penetration, and higher education levels. A typical example is the “Yahoo Boys” in Nigeria, referring to young Nigerians engaged in cyber fraud through Yahoo mail, mostly well-educated undergraduates with digital skills (Lazarus and Okolorie, 2019). A range of factors, such as a high rate of unemployment, a lack of legitimate economic opportunities, a prevalence of cybercrime subculture, a lack of strong cybercrime laws, and a high level of corruption, have motivated them to obtain illegal wealth through cybercrime. In contrast, cybercrime in high-income regions originates in areas with a high Gini index and a high education level. One possible explanation for this finding may be that well-educated individuals who live in countries with a high Gini index are paid less for their skills than their counterparts, which motivates them to engage in cybercrimes to improve their lives.

Encouragingly, both the GLM and SEM results suggest that political factors and cybersecurity preparedness can mitigate the incidence of cybercrime to some extent, in agreement with the hypotheses. Though previous country-level studies suggest that countries facing more cybersecurity threats tend to have a high level of cybersecurity preparedness (Makridis and Smeets, 2019; Calderaro and Craig, 2020), our results indicate that cybersecurity preparedness could in turn reduce cybercrimes that originate from a country. This emphasises the importance of government intervention and cybersecurity capacity building. The necessary intervening measures may include the enactment and enforcement of laws, regulation of telecommunication operators and internet service providers (ISPs), strengthening of strike force by security and judicial departments, and improvement of cybersecurity capacity. Given the interconnectedness of cyberspace and the borderless nature of cybercrime, it must be recognised that cybersecurity is not a problem that can be solved by any single country. Thus, enhancing international cooperation in legal, technical, organisational, and capacity aspects of cybersecurity becomes an essential way to tackle cybersecurity challenges.

As presented through SEM, technological factors are closely associated with the development of socioeconomic development and serve as a mediator between socio-economic conditions and cybercrime. In the past decades, ICTs have developed unevenly across different parts of the world due to a range of geographic, socioeconomic, and demographic factors, which has led to the global digital divide (Pick and Azari, 2008). The disparities in internet access in different regions have largely determined the spatial patterns of cybercrime. Currently, developing countries (especially those within Asia, Africa, and Latin America) are the fastest-growing regions in terms of ICT infrastructure and internet penetration (Pandita, 2017). However, even in developed countries, the progress of technological innovation has outpaced the establishment of legal regulations, national institutions and frameworks, policies and strategies, and other mechanisms that could help manage the new challenges (Bastion and Mukku, 2020). Many developing countries are facing difficulties in combating cybercrime due to a lack of adequate financial and human resources, legal and regulatory frameworks, and technical and institutional capacities, providing a fertile ground for cybercrime activities. In this vein, it is extremely urgent and necessary to enhance the cybersecurity capacities of developing countries and engage them in the international cooperation of cybersecurity, ensuring that they can maximize the socio-economic benefits of technological development instead of being harmed by it.

Cybercrime is a sophisticated social phenomenon rooted in deep and comprehensive geographical and socioeconomic causes. This study offers an alternative perspective in solving cybersecurity problems instead of pure technical measures. We believe that improvements in cybersecurity require not only technological, legal, regulatory, and policing measures but also broader approaches that address the underlying social, economic, and political issues that influence cybercrime. While the results presented in this study are preliminary, we hope that this work will provide an extensible framework that can be expanded for future studies to investigate the driving forces of cybercrime.

However, our study has several limitations due to the disadvantages of data. First and foremost, the geo-localisation of cybercrimes or cybercriminals remains a major challenge for cybercrime research. Although the FireHOL IP blocklist has the potential to measure global cybercrime at a high spatial resolution, IP-based measures may not accurately capture the true locations of cybercriminals, as they may simply exploit places with better ICT infrastructure. Therefore, caution should be exercised in interpreting the associations between cybercrime and socioeconomic factors. Future studies combining survey data, police and court judgement data, and cybercrime attribution techniques are needed to further validate the accuracy and validity of IP-based technical data in measuring the geography of cybercrime and gain a deeper understanding of the driving forces of cybercrime. Besides, COVID-19 has greatly changed the way we live and work, and many studies have suggested that the pandemic has increased the frequency of cybercrimes within the context of economic recession, high unemployment, accelerated digital transformation, and unprecedented uncertainty (Lallie et al., 2021; Eian et al., 2020; Pranggono and Arabo, 2021). Unfortunately, the blocklist data cannot well capture this dynamic due to a lack of temporal attributes. Furthermore, different types of cybercrime can be influenced by different mechanisms. We use the total amount of all types of cybercrime IPs instead of looking into a specific type of cybercrime, given that such segmentation may result in data sparsity for some groups. Future studies are needed to determine how different categories of cybercrimes are affected by socioeconomic factors. At last, micro-level individual and behaviour characteristics and more fine-grained explanatory variables should be included to better understand cybercrime.

## Data Availability

The FireHOL IP lists data are publicly available at the FireHOL website (https://iplists.firehol.org/ and https://github.com/firehol/blocklist-ipsets); population, education index, income index, HDI, and subnational regions data are available from Global Data Lab (https://globaldatalab.org); nighttime light data are available from the Earth Observation Group (https://eogdata.mines.edu/download_dnb_composites.html); Population aged 15–64, Gini index, GDP growth, unemployment, poverty rate, control of corruption, government effectiveness, rule of law, political stability and absence of violence/terrorism, and voice and accountability, are obtained from World Bank (https://databank.worldbank.org/home.aspx), the internet users, international bandwidth, secure internet server, and fixed broadband subscriptions are available from International Telecommunication Union (ITU) (https://www.itu.int/itu-d/sites/statistics); the internet infrastructure are collected from TeleGeography (https://www.internetexchangemap.com) and the World Data Centers Database (https://datacente.rs); the legal measures, technical measures, organisational measures, capacity development, cooperation measures and overall cybersecurity index were obtained from the Global Cybersecurity Index (GCI) of the ITU (https://www.itu.int/en/ITU-D/Cybersecurity/Pages/global-cybersecurity-index.aspx).
